# Roles of *orf60a* and *orf61* in Development of Bacteriophages λ and Φ24_B_

**DOI:** 10.3390/v10100553

**Published:** 2018-10-11

**Authors:** Aleksandra Dydecka, Bożena Nejman-Faleńczyk, Sylwia Bloch, Gracja Topka, Agnieszka Necel, Logan W. Donaldson, Grzegorz Węgrzyn, Alicja Węgrzyn

**Affiliations:** 1 Department of Molecular Biology, Faculty of Biology, University of Gdańsk, Wita Stwosza 59, 80-308 Gdańsk, Poland; aleksandra.dydecka@phdstud.ug.edu.pl (A.D.); bozena.nejman@biol.ug.edu.pl (B.N.-F.); sylwia.bloch@biol.ug.edu.pl (S.B.); gracja.topka@phdstud.ug.edu.pl (G.T.); agnieszka.necel@phdstud.ug.edu.pl (A.N.); 2 Department of Biology, York University, 4700 Keele Street, Toronto, ON M3J 1P3, Canada; logand@yorku.ca; 3 Laboratory of Molecular Biology, Institute of Biochemistry and Biophysics, Polish Academy of Sciences, Kładki 24, 80-822 Gdańsk, Poland; alicja.wegrzyn@biol.ug.edu.pl

**Keywords:** lambdoid phages, Shiga toxin-converting bacteriophage, regulation of bacteriophage development, the *exo-xis* region

## Abstract

The *exo-xis* region of lambdoid bacteriophage genomes contains several established and potential genes that are evolutionarily conserved, but not essential for phage propagation under laboratory conditions. Nevertheless, deletion or overexpression of either the whole *exo-xis* region and important regulatory elements can significantly influence the regulation of phage development. This report defines specific roles for *orf60a* and *orf61* in bacteriophage λ and Φ24_B_, a specific Shiga toxin-converting phage with clinical relevance. We observed that mutant phages bearing deletions of *orf60a* and *orf61* impaired two central aspects of phage development: the lysis-*versus*-lysogenization decision and prophage induction. These effects were more pronounced for phage Φ24_B_ than for λ. Surprisingly, adsorption of phage Φ24_B_ on *Escherichia coli* host cells was less efficient in the absence of either *orf60a* or *orf61*. We conclude that these open reading frames (ORFs) play important, but not essential, roles in the regulation of lambdoid phage development. Although phages can propagate without these ORFs in nutrient media, we suggest that they may be involved in the regulatory network, ensuring optimization of phage development under various environmental conditions.

## 1. Introduction

Bacteriophage λ, and other bacteriophages of the lambdoid class that share a similar genome organization and life cycle, have contributed to many important discoveries in molecular biology, from the regulation of gene expression and DNA replication to macromolecular interactions and novel biological structures [1,2,3]. Some lambdoid phages carry genes encoding toxins that function as virulence factors for several species of pathogenic bacteria. Examples of such bacteria are Shiga toxin-producing *Escherichia coli* (STEC) strain and enterohemorrhagic *E. coli* (EHEC) associated with food poisoning outbreaks often accompanied by fatalities in immune-compromised individuals [4,5,6]. As a result, a precise understanding of the developmental regulation of lambdoid phages is important not only for basic science, but also for identifying new leads for drug discovery.

**Figure 1:**
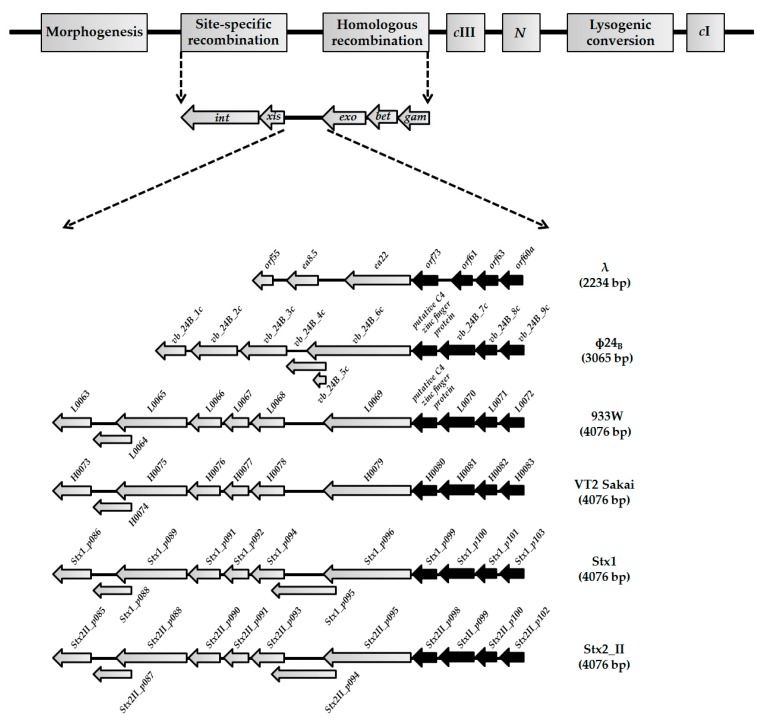
Genes and open reading frames (ORFs) located between *exo* and *xis* genes in genomes of lambdoid bacteriophages: λ (NC_001416), Φ24_B_ (HM208303), 933W (NC_000924), VT2 Sakai (AP000422), Stx1 (NC_004913), and Stx2_II (NC_004914). Black arrows represent highly conserved *orf60a*–*orf73* regions among lambdoid phages (≥70% nucleotide sequence identity). Grey arrows with black borders present genes and ORFs with lower level of identity (<35%) or additional ORFs that occur in the *exo-xis* regions of Stx bacteriophages.

Viral genomes may be considered as compact systems bearing genes that are most necessary and optimized for propagation. This is due to the evolutionary pressure to select minimal genomes that are easy to replicate, and can be effectively packaged into capsids. It is surprising, therefore, that non-essential genes in λ and related phages are still observed. One such region (*exo-xis*) is found between the well characterized *exo* and *xis* genes, and is comprised of several potential open reading frames (ORFs). While the *exo-xis* region is dispensable for propagation of lambdoid bacteriophages under laboratory conditions [7], it is evolutionarily conserved which implicates its possible important role in bacteriophage development [8] (Figure 1).

Since the *exo-xis* region did not appear to markedly affect lytic development during the first studies, any defined roles for its genetic elements and functional insights into any of gene products were not intensively investigated, and remained almost completely unknown for a considerable period of time. Approximately ten years ago, our team has demonstrated that lysogenization of *E. coli* cells by phage λ was impaired when the *exo-xis* region was overexpressed [9]. Under similar conditions, induction of prophages λ and Φ24_B_ was found to be more effective [8,10]. The Ea8.5 protein, encoded in this region, appeared to play important role in this regulation [9,10], and detailed structural studies revealed that it contains a fused homeodomain/zinc-finger fold, suggesting a potential regulatory function [11]. Intriguingly, when induction of prophages λ and Φ24_B_ was provoked by various agents (mitomycin C or hydrogen peroxide), different expression patterns of genes from the *exo-xis* region were observed [10]. This suggested that genes and ORFs from this region may be involved in regulatory processes occurring under, and responding to, various environmental conditions. Oxidative stress appeared to be predominant environmental condition influencing *exo-xis* mediated phage development [12]. Therefore, studies on particular genes and ORFs from this region appeared substantiated. Recently, we demonstrated that deletion of *orf63* resulted in delayed and less efficient induction of λ and Φ24_B_ prophages. Since this ORF encodes a structured protein, it follows that *orf63* is a functional gene [13].

In this study of the *exo-xis* region, we have concentrated on the functions of *orf60a* and *orf61* to obtain the first insights regarding their roles in the regulation of development of lambdoid phages. To make this work compatible with previous reports, λ bacteriophage and the clinically relevant Φ24_B_ bacteriophage were used in this study.

## 2. Materials and Methods

### 2.1. Bacterial Strains and Bacteriophages and Plasmids

*E. coli* MG1655 strain, its derivatives, and bacteriophages used in this work, are listed in Table 1.

The deletion mutants were constructed as described previously [12,14] by using *E. coli* MG1655 (λ) or *E. coli* MG1655 (Φ24_B_) strains. The procedure was performed according to the manufacturer’s protocol of the Quick and Easy *E. coli* Gene Deletion Kit (Gene Bridges, Heidelberg, Germany). In the first step, the nucleotide sequence of *orf60a* or *orf61* has been replaced with FRT-flanked kanamycin resistance cassette. Then, the selection marker was removed in the FLP-recombinase step, leaving 87 nucleotides of the cassette in the place of the original sequence of *orf60a* or *orf61* in the genome of lysogenic *E. coli* bacteria. All constructs (phage genomes with deletions of either *orf60a* or *orf61*) were confirmed by DNA sequencing.

Bacteriophage suspensions were routinely stored in Tris-HCl-Magnesium sulfate buffer (TM buffer; 10 mM Tris-HCl, 10 mM MgSO_4_, pH 7.2) at 4 °C. *E. coli* MG1655 strain was selected as a host for bacteriophage infection. Bacteria were cultured in the Luria-Bertani (LB) medium at 30 °C.

### 2.2. Prophage Induction Experiments

Bacteria lysogenic with tested phages were grown in LB medium at 30 °C, with a shaking, until the OD_600_ reached 0.1, and then treated with 0.2 µg/mL mitomycin C or 1 mM hydrogen peroxide to induce prophages. Following the induction step, at indicated times (every 30 min), 0.5 mL samples were withdrawn, mixed with chloroform, and centrifuged (2000× *g* for 5 min at room temperature). Afterwards, serial dilutions were prepared in TM buffer and 2.5 μL of each dilution was spotted onto plates with double-layer LB agar (phage λ) or LB agar supplemented with 2.5 μg/mL chloramphenicol (Φ24_B_ phage), that were prepared according to a procedure described previously [18]. A separate set of analogous experiments with each lysogenic strain was performed without addition of the induction agent. Such control experiments allowed for estimating the levels of spontaneous prophage induction. Following an overnight incubation of plates at 37 °C, the relative phage titer (PFU/mL) was determined by subtracting the phage titer of the non-induced culture from the phage titer of a respective induced variant.

### 2.3. One-Step Growth Experiment

To examine the intracellular life cycle of analyzed phages, a procedure described previously [10,14], was employed, with a few minor modifications. Briefly, bacteria were grown in LB medium at 30 °C, with shaking, until the OD_600_ reached 0.2. At this stage, 10 mL of bacterial culture was centrifuged (2000× *g* for 10 min at 4 °C) and the obtained pellet was suspended in 1 mL of LB enriched with 3 mM NaN_3_ (Sigma-Aldrich, St. Louis, MO, USA). Following a 5 min incubation at 30 °C, the phage lysate was added to an multiplicity of infection (m.o.i.) of 0.05, and then incubated again for 10 min. Afterwards, the sample was washed 3 times with LB supplemented with 3 mM NaN_3_, and centrifuged each time (3000× *g* for 10 min at 4 °C). After unadsorbed phages were removed, 250 μL of the suspension was added to 25 mL of LB medium prewarmed to 30 °C (time 0), and cultivated at this temperature with shaking. To estimate the number of infection centers, 0.2 mL culture samples were collected at 5, 10, and 15 min post-infection, and mixed with 0.8 mL indicator bacteria and 2 mL 0.7% top agar (prewarmed to 45 °C), supplemented with MgSO_4_ (phage λ) or MgSO_4_ and CaCl_2_ (phage Φ24_B_), to a final concentration of 10 mM each. The mixtures were then poured onto LB plates (phage λ) or LB plates enriched with 2.5 μg/mL chloramphenicol (phage Φ24_B_). The phage titer was determined by collecting 0.5 mL samples that were prepared by shaking in a chloroform mixture followed by centrifugation (2000× *g* for 5 min). Phage lysates from this step were diluted in TM buffer, and titrated under permissive conditions. Following an overnight incubation at 37 °C, the burst size (number of virions released from single infected cell) was estimated as a ratio of a phage titer to the titer of infection centers.

### 2.4. Measurement of the Efficiency of Phage Adsorption

Bacteria were grown in LB medium at 30 °C, with shaking, until the OD_600_ reached 0.1, upon which samples of 1 mL were centrifuged (2000× *g* for 10 min at 4 °C) and the pellets subsequently dissolved in 0.15 mL of 0.85% NaCl. This mixture was centrifuged (2000× *g* for 10 min at 4 °C) and pellets were suspended in 0.15 mL LB medium supplemented with 10 mM MgSO_4_ (phage λ) or 10 mM MgSO_4_ + 10 mM CaCl_2_ (phage Φ24_B_). Following a 15 min incubation at 30 °C, tested bacteriophages were added to an m.o.i. of 0.1, and such mixtures were incubated at 30 °C. At specified times after the addition of phage lysate, the samples were withdrawn, centrifuged in a microcentrifuge (12,000× *g* for 1 min at room temperature) and supernatants were titrated on indicator *E. coli* bacteria. Plates were incubated at 37 °C overnight. In this method, the efficiency of phage adsorption was estimated by using the ln (*P_t_*/*P*_0_) equation described previously [19], where *P_t_* and *P*_0_ are phage concentrations at indicated times and time zero (immediately after addition of bacteriophages), respectively.

### 2.5. Efficiency of Lysogenization

Host *E. coli* bacteria were grown in LB medium at 30 °C, with shaking, until the OD_600_ reached 0.2. Next, 1 mL of culture was centrifuged (2000× *g* for 10 min at room temperature), and the obtained pellet was dissolved in 1 mL of TM buffer and, again, centrifuged (2000× *g* for 5 min at room temperature) and suspended. Following a short incubation at 30 °C, bacteriophages were added to an m.o.i. of 1, 5, and 10 and, next, the mixtures were again incubated at 30 °C. Serial dilutions were prepared in TM buffer and 40 μL of each dilution was poured onto LB plates. After overnight incubation at 37 °C, 96 colonies were passaged separately each in a well of a 96-well plate with 200 μL of LB medium. Each plate was shaken at 37 °C until the OD_600_ reached 0.1 and irradiated with UV light at 50 J/m^2^ for 20 seconds to induce prophages, followed by an incubation at 37 °C for 1 h. After induction, lysogens were mixed with chloroform, centrifuged (2000× *g* for 10 min at 4 °C), and 2.5 μL of each top layer was spotted on a freshly prepared plate with double layer LB agar (phage λ) or LB agar supplemented with 2.5 μg/mL chloramphenicol (phage Φ24_B_). Plates were incubated at 37 °C overnight. The efficiency of lysogenization was determined as a percent of lysogens among all tested 96 bacterial colonies. Each experiment was repeated three times. Lysogens were also verified by testing their resistance to superinfection, as indicated previously [20].

### 2.6. Survival of Cells after Bacteriophage Infection

To determine the survival of the wild-type *E. coli* strain after phage infection, a previously published method [13,21] was used with only minor modifications. Briefly, bacteria were grown in LB medium at 30 °C, with shaking, until the OD_600_ reached 0.2. Following centrifugation (2000× *g* for 10 min at 4 °C), pellets were washed with 0.85% NaCl, and then suspended in 1.2 mL of LB medium enriched with 10 mM MgSO_4_ (phage λ) or 10 mM MgSO_4_ + 10 mM CaCl_2_ (phage Φ24_B_). Next, samples were incubated for 15 min at 30 °C, and tested bacteriophages were added to an m.o.i. of 1, 5 or 10. Following another incubation at 30 °C, serial dilutions of the initial samples were prepared in 0.85% NaCl, and 40 μL of each dilution was plated on LB agar plates and incubated at 37 °C overnight. The number of viable bacterial cells was calculated on the basis of counted colonies. The fraction of surviving bacterial cells in a population infected with the tested phages was calculated in relation to the control experiment, in which TM buffer was used instead of phage lysate.

### 2.7. Measurement of Bacterial Viability during Prophage Induction Experiments

Bacterial viability was measured following a published procedure that is briefly described here [10]. *E. coli* lysogenic with tested phages was grown in LB medium at 30 °C, with shaking, until the OD_600_ reached 0.1, and then treated with 1 mM hydrogen peroxide as an induction agent. At various times post-induction, samples of 2 × 10^8^ cells/mL were withdrawn and centrifuged (8000× *g* for 10 min at room temperature). Pellets were washed and suspended in 0.85% NaCl. Samples prepared were stained with LIVE/DEAD *Bac*Light Bacterial Viability Kit (Molecular Probes, Eugene, OR, USA), which provides an estimate of live bacteria under the assumption they have intact cell membranes. Following the manufacturer’s protocol, fluorescence measurements were performed in a microplate reader using an excitation wavelength of 485 nm and emission wavelengths of 530 and 630 nm. Presented values indicate the percent of live bacteria normalized to results of control experiments (cultures without induction agent) which, at each time, were assumed as 100% live bacteria.

## 3. Results

### 3.1. Sequences of orf60a and orf61, and Their Putative Products, Are Conservative among Lambdoid Phages

Since the *exo-xis* region presents a similar organization and sequence conservation among lambdoid phages [8,10,12,13], we began by determining if sequences of *orf60a* and *orf61*, as well as putative products of these ORFs, were similar among phages from this group. We observed that scores of pairwise nucleotide alignments of *orf60a* indicate generally high similarities (>90% identity) between this ORF and six lambdoid phages (Table 2).

A similar analysis of the predicted amino acid sequences of the putative Orf60a protein demonstrated a high degree of similarity among tested phages (>90% identity) (Table 3).

Analogous analyses of *orf61* indicated even higher similarities of both nucleotide and amino acid sequences. For this ORF, and its putative product, nucleotide, and amino acids similarities were high for all six tested phages (Table 4 and Table 5, respectively). From these comparisons, it is possible that *orf60a* and *orf61* are true genes that encode functional proteins (Table 3 and Table 5, respectively).

### 3.2. Influence of orf60a and orf61 on Prophage Induction with Various Inductors

**Figure 2:**
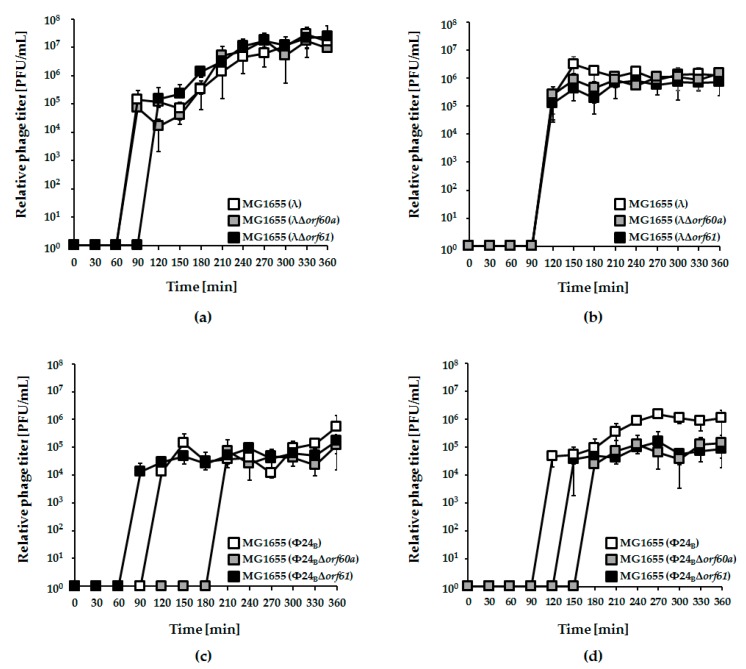
Development of bacteriophages λ (panels (**a**) and (**b**)) and Φ24_B_ (panels (**c**) and (**d**)), either wild-type (white squares), Δ*orf60a* (grey squares), or Δ*orf61 (*black squares) after induction of lysogenic *Escherichia coli* MG1655 strain with 0.2 µg/mL mitomycin C (panels (**a**) and (**c**)) or 1 mM hydrogen peroxide (panels (**b**) and (**d**)). The presented results are mean values from three independent experiments (biological samples), with error bars indicating the standard deviation (S.D.).

Either deletion or overexpression of the *exo-xis* region affects the induction of lambdoid prophages [8,10,12,13]. Here, we have tested if specific deletions of *orf60a* or *orf61*, alone, can alter induction of λ and Φ24_B_ prophages by mitomycin C or hydrogen peroxide (Figure 2).

**Figure 3:**
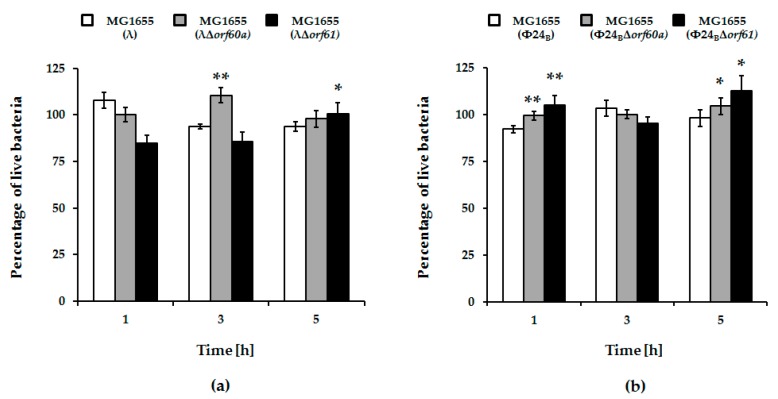
Percentage of live *E. coli* MG1655 cells lysogenic with λ (panel (**a**)) and Φ24_B_ (panel (**b**)), either wild-type (white columns), Δ*orf60a* (grey columns), or Δ*orf61* (black columns) during prophages induction with 1 mM hydrogen peroxide. The presented results are mean values from three biological experiments with error bars indicating S.D. Statistical analysis was performed by using Student’s *t*-test. Significant differences between fractions of bacterial cells lysogenic with wild-type phages and their deletion mutants are marked by asterisks, *p* < 0.05 (*) or *p* < 0.01 (**).

Irrespective of the kind of the inductor used in experiments, deletion of either *orf60a* or *orf61* had only minor effects on induction of λ prophage and phage development (Figure 2, panels a and b). For Φ24_B_, however, the effects were more dramatic. Induction of the Φ24_B_Δ*orf60a* prophage by both mitomycin C and hydrogen peroxide was significantly delayed, and the efficiency of lytic development was lower, relative to wild-type phage (Figure 2, panels c and d). Therefore, *orf60a* appears to play a significant role in the control of prophage induction. In accordance with the induction experiments, we have also observed higher survival of *E. coli* host cells after induction of mutant Φ24_B_ prophages, relative to their wild-type counterparts (Figure 3). Although an opposite trend was observed at time 1 h in experiments with λ and at time 3 h with Φ24_B_, these differences were not statistically significant (Figure 3), thus, we conclude as described above.

### 3.3. Effects of orf60a and orf61 Deletions on Phage Infection

**Figure 4:**
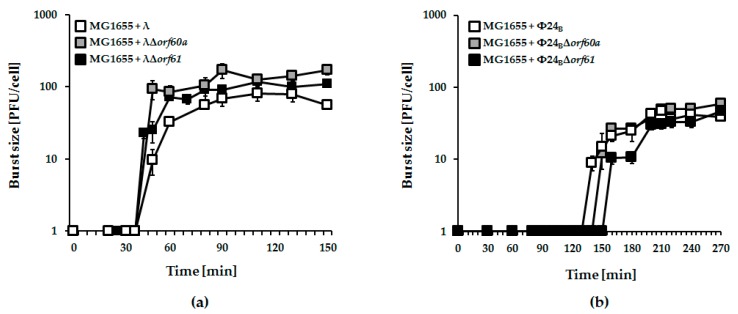
One-step growth experiments with λ (panel (**a**); white squares), Φ24_B_ (panel (**b**); white squares), and recombinant phage mutants bearing deletions of *orf60a* (panels (**a**) and (**b**); grey squares) or *orf61* (panels (**a**,**b**); black squares), infecting *E. coli* MG1655 host. Mean values from three independent experiments with error bars indicating S.D. are shown.

Next, we tested the effects of *orf60a* or *orf61* deletions on infection of host cells by λ and Φ24_B_ phages. In one-step growth experiments, no significant effects were observed (Figure 4).

**Figure 5:**
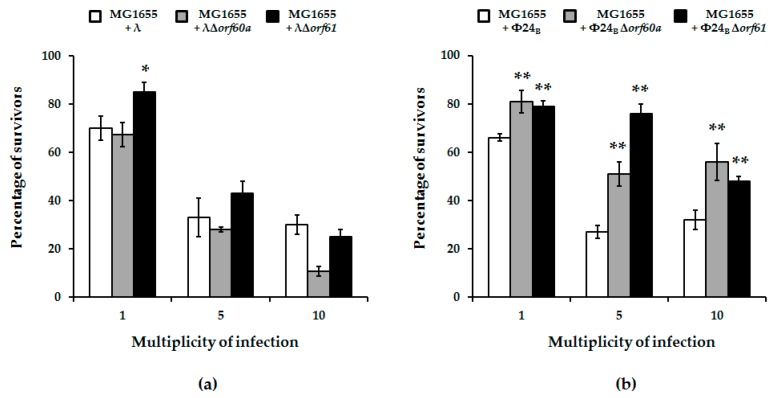
Percentage of survivors of *E. coli* MG1655 bacteria after infection of wild-type phages: λ (panel (**a**); white columns) and Φ24_B_ (panel (**b**); white columns) or their deletion mutants: λΔ*orf60a* (panel (**a**); grey columns), λΔ*orf61* (panel (**a**); black columns), Φ24_B_Δ*orf60a* (panel (**b**); grey columns), and Φ24_B_Δ*orf61* (panel (**b**); black columns). Results are presented as mean values ± S.D. from three biological experiments. A *t*-test was performed for results from each multiplicity of infection (m.o.i.). The significance of differences between fractions of bacterial cells infected with wild-type phages and their deletion mutants are marked by asterisks, *p* < 0.05 (*) or *p* < 0.01 (**).

However, when post-infection host survival was assessed, it was significantly higher for mutant Φ24_B_ phages (either devoid of *orf60a* or *orf61*) relative to wild-type phage (Figure 5; panel b). Again, similar deletions tested with phage λ were less pronounced (Figure 5; panel a).

**Figure 6:**
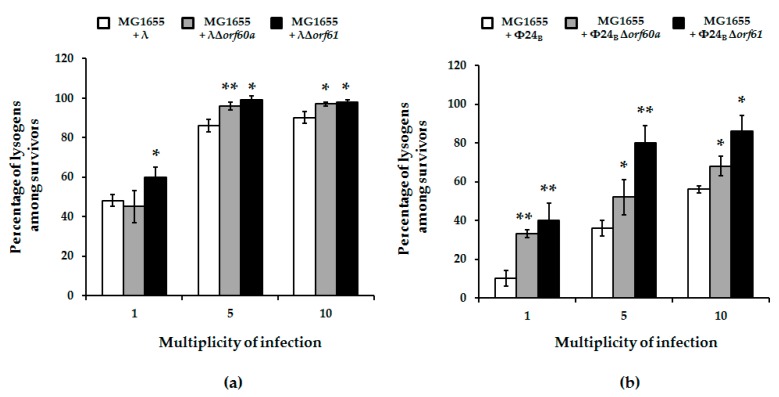
Fraction of lysogens among survivors of *E. coli* MG1655 bacteria after infection with wild-type bacteriophages: λ (panel (**a**); white columns) and Φ24_B_ (panel (**b**); white columns) or their deletion mutants: λΔ*orf60a* (panel (**a**); grey columns), λΔ*orf61* (panel (**a**); black columns), Φ24_B_Δ*orf60a* (panel (**b**); grey columns), and Φ24_B_Δ*orf61* (panel (**b**); black columns). Results are presented as mean values ± S.D. from three independent experiments. A *t*-test was performed for results from each m.o.i. Statistically significant differences between wild-type phage and its deletion mutants are marked by asterisks, *p* < 0.05 (*) or *p* < 0.01 (**).

To explore possible causes for enhanced bacterial survival upon mutant phage infection, we assessed the efficiency of lysogenization on host cells. Formation of lysogenes was more effective in the absence of *orf60a* or *orf61*. This was true for both λ and Φ24_B_ phages, but the effects were more pronounced in the case of Φ24_B_ (Figure 6). We therefore conclude that these ORFs have regulatory roles that determine the decision to enter the lytic or lysogenic stage of bacteriophage development.

### 3.4. Adsorption of Phage Φ24_B_ is Impaired in the Absence of orf60a or orf61

**Figure 7:**
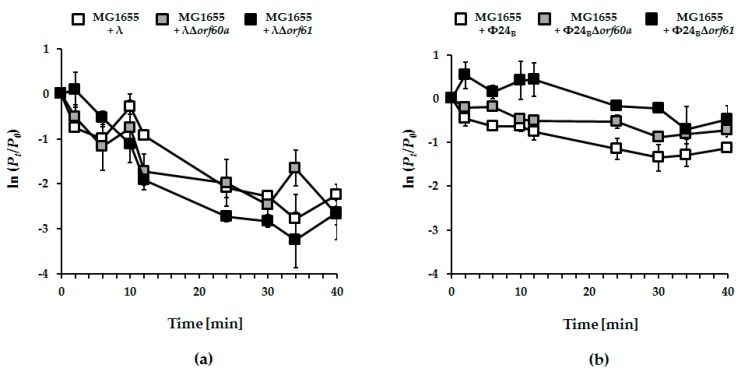
Kinetics of adsorption of lambdoid bacteriophages: λ (panel (**a**); white squares), Φ24_B_ (panel (**b**); white squares) and their mutants bearing deletions of *orf60a* (panels (**a**) and (**b**); grey squares) or *orf61* (panels (**a**) and (**b**); black squares) on *E. coli* MG1655 host. Tested bacteriophages were added to bacterial cell suspension to m.o.i. of 0.1. Ratios of unadsorbed bacteriophages were logarithmically transformed according to the ln (*P_t_*/*P*_0_) equation, where *P_t_* and *P*_0_ are phage concentrations at tested times and time zero, respectively. The presented results are mean values from three biological experiments with S.D. indicated by error bars.

The earliest stages of infection depend on efficient adsorption of phage on the host cells. No significant effects of deletion of *orf60a* or *orf61* could be found in phage λ (Figure 7; panel a). However, adsorption of Φ24_B_ phages devoid of either *orf60a* or *orf61* was significantly impaired (Figure 7; panel b). Together, the results suggest that Orf60a and Orf61 proteins may be involved in the process of the virion assembly, with impairment of this process leading to the formation of partially defective virions that are less effective at adsorbing onto host cells.

## 4. Discussion

While lambdoid bacteriophages have been studied for decades, it is remarkable that there are still many genes, particularly in the *exo-xis* region, whose roles remain unknown. Given that many genes are evolutionarily conserved, it stands to reason that many, if not all, serve important functions in phage development. Likewise, the *exo-xis* genes also then present several paths towards the development of new therapeutics that target pathogenic/toxicogenic bacteria that are lysogenic for phages like Φ24_B_.

In this report, we have continued an investigation of genes in the *exo-xis* region. Previous studies demonstrated that this region, although not essential for phage lytic propagation under laboratory conditions, was still important for precise control of some steps of phage development [7,8,9]. The Ea8.5 protein, for example, participates in the control of gene expression, and its structure suggests regulatory functions [10,12,13]. The *orf63* gene also encodes a protein, and appears to control prophage induction and regulation of expression of genes from the *exo-xis* region [13]. In this work, we investigated effects of deletions of *orf60a* and *orf61* on development of phage and Shiga toxin-converting phage Φ24_B_. Our results indicate that these ORFs significantly influence efficiency and timing of prophage induction, as well as the efficiency of lysogenization. Interestingly, differences between mutant and wild-type phages were more pronounced in Φ24_B_ than in λ. When analyzing results of experiments performed to assess efficiency of lysogenization, one might argue that higher fractions of lysogens detected among bacterial cells which survived infection with *orf60a* and *orf61* mutants in comparison to wild-type phages (Figure 6) might result from higher effectivity of adsorption on host cells or less efficient lytic development, rather than actual differences in efficiency of forming prophages. However, contrary to such predictions, we have demonstrated that the mutant phages adsorb on *E. coli* cells less efficiently than wild-type viruses (Figure 7), and their lytic development is not impaired (Figure 4). Therefore, we propose that lysogenization is more effective in the absence of functional *orf60a* and *orf61* regions.

Although detailed molecular mechanisms of functions of *orf60a* and *orf61* products remain to be elucidated, we speculate that they are involved in the control of gene expression. Our attempts to investigate structures of products of *orf60a* and *orf61* were, as yet, unsuccessful, due to problems with obtaining milligram quantities of soluble folded proteins that were suitable for biochemical and structural studies. However, the observed effects of deletions of these ORFs suggest that they might potentially participate in regulatory networks controlling prophage induction and the lysis-versus-lysogenization decision. Although we could not demonstrate directly that effects of dysfunctions of *orf60a* and *orf61* are due to expression of diffusible gene products (overexpression of these ORFs from a plasmid resulted in production of insoluble protein aggregates), there are some suggestions that these genetic elements code for real protein products. The presence of *orf60a-* and *orf61-*derived transcripts could be detected by RT-qPCR [10]. Since amounts of such transcripts were different under various growth conditions and at various times after initiation of phage lytic development [10], one might suggest that the expression levels of *orf60a* and *orf61* can be of importance in modulation of some regulatory processes. Moreover, when λ ORFs were cloned into Gateway vectors, and protein–protein interactions were tested in the yeast two-hybrid system, interactions between the product of *orf61* and those of *int* and *orf-314* were detected [22]. Then, experiments with ribosome profiling, aimed to study the network of proteins encoded by bacteriophage λ, indicated the presence of *orf60a* and *orf61* protein products in *E. coli* cells after prophage induction in the lysogenic host [23]. Therefore, one may conclude that these ORFs code for specific proteins. We are aware that still this is not a proof that effects observed in our experiments with mutants in *orf60a* and *orf61* were due to the lack of appropriate diffusible molecules. Nevertheless, although we cannot exclude that deletion of either *orf60a* or *orf61*, or both, could cause changes in the phage genome structure or alter expression of other genes, which would result in changes of bacteriophage developed we observed in our experiments, we suggest that, in the light of results presented in this paper, as well as in previously published articles [10,22,23] demonstrating actual expression of both tested ORFs at levels of RNAs and proteins, the hypothesis that *orf60a* and *orf61* code for functional proteins is more likely.

One of the most intriguing aspects of our study on the effects *orf60a* or *orf61* deletion was observed at the adsorption phase of Φ24_B_ phage on host cells. One possible hypothesis leading from this observation is that putative products of these ORFs are involved in the control of either expression of particular genes coding for capsid proteins, or participate directly in macromolecular interactions at critical stages during the formation of functional virions. This hypothesis is supported by observations that virions of Φ24_B_ (as well as some other Shiga toxin-converting phages) are significantly more sensitive to UV irradiation than bacteriophage λ, most probably due to the less stable structure of the virion of the former phage which is, thus, more susceptible for damage [24]. If so, precise control of the virion assembly might be particularly important for Φ24_B_, and any disturbance in this process might result in significant loss of some functions, like adsorption on host cells.

## 5. Conclusions

In this study of *exo-xis* region of lambdoid phages, we have observed that deletions of *orf60a* and *orf61* dysregulate prophage induction and alter the lysis-versus-lysogenization decision of phages λ and Φ24_B_. Impaired adsorption of virions on host cells was also observed, and specific to phage Φ24_B_. An explanation for these effects, possibly related to altered gene expression or macromolecular interactions, will require further experiments with purified and active Orf60a and Orf61 proteins.

## Figures and Tables

**Table 1:** Bacterial strains and bacteriophages.

Bacterial Strains or Bacteriophages	Relevant Genotype or Description	References
***Escherichia coli* Strains**		
MG1655	F^–^ λ^–^ *ilvG rfb-50 rph-1*	[15]
MG1655 (λ)	MG1655 bearing λ prophage	[8]
MG1655 (λΔ*orf60a*)	MG1655 bearing λ prophage with deletion of *orf60a*	[12]
MG1655 (λΔ*orf61*)	MG1655 bearing λ prophage with deletion of *orf61*	[12]
MG1655 (Φ24_B_)	MG1655 bearing Φ24_B_ prophage	[8]
MG1655 (Φ24_B_Δ*orf60a*)	MG1655 bearing Φ24_B_ prophage with deletion of *vb_24B_9c*, the homolog of λ*orf60a*	[12]
MG1655 (Φ24_B_Δ*orf61*)	MG1655 bearing Φ24_B_ prophage with deletion of *vb_24B_7c*, the homolog of λ*orf61*	[12]
**Bacteriophages**		
λ	carries a frameshift mutation relative to Ur-lambda	[16]
λΔ*orf60a*	λ phage with deletion of *orf60a*	[12]
λΔ*orf61*	λ phage with deletion of *orf61*	[12]
Φ24_B_	Φ24_B_s*tx2*::*cat*	[17]
Φ24_B_Δ*orf60a*	Φ24_B_ phage with deletion of *vb_24B_9c*, the homolog of Φ24_B_ *orf60a*	[12]
Φ24_B_Δ*orf61*	Φ24_B_ phage with deletion of *vb_24B_7c*, the homolog of Φ24_B_ *orf61*	[12]

**Table 2:** Scores of pairwise alignments of the nucleotide sequences of *orf60a* from six analyzed lambdoid phages: λ phage (NC_001416), Φ24_B_ phage (HM208303), 933W phage (NC_000924), VT2 Sakai phage (AP000422), Stx1 converting phage (NC_004913), and Stx2 converting phage II (NC_004914).

	λ	Φ24_B_	933W	VT2 Sakai	Stx1	Stx2_II
**λ**		96	96	94	94	94
**Φ24_B_**			100	94	94	94
**933W**				94	94	94
**VT2 Sakai**					100	100
**Stx1**						100
**Stx2_II**						

The multiple sequence alignment was performed using the ClustalW algorithm. Pairwise scores represent the percentage identity between two sequences, taking into account the length of the alignment.

**Table 3:** Scores of pairwise alignments of the predicted amino acid sequences of Orf60a from six analyzed lambdoid phages: λ phage (NC_001416), Φ24_B_ phage (HM208303), 933W phage (NC_000924), VT2 Sakai phage (AP000422), Stx1 converting phage (NC_004913), and Stx2 converting phage II (NC_004914).

	λ	Φ24_B_	933W	VT2 Sakai	Stx1	Stx2_II
**λ**		95	95	97	97	97
**Φ24_B_**			100	94	94	94
**933W**				94	94	94
**VT2 Sakai**					100	100
**Stx1**						100
**Stx2_II**						

Pairwise scores are simply the number of identities between the two sequences, divided by the length of the alignment, and represented as a percentage. The multiple sequence alignment was performed using the ClustalW algorithm.

**Table 4:** Scores of pairwise alignments of the nucleotide sequences of *orf61* from six analyzed lambdoid phages: λ phage (NC_001416), Φ24_B_ phage (HM208303), 933W phage (NC_000924), VT2 Sakai phage (AP000422), Stx1 converting phage (NC_004913), and Stx2 converting phage II (NC_004914).

	λ	Φ24_B_	933W	VT2 Sakai	Stx1	Stx2_II
**λ**		80	80	80	80	80
**Φ24_B_**			100	100	100	100
**933W**				100	100	100
**VT2 Sakai**					100	100
**Stx1**						100
**Stx2_II**						

Pairwise scores are simply the number of identities between the two sequences, divided by the length of the alignment, and represented as a percentage. The multiple sequence alignment was performed using the ClustalW algorithm.

**Table 5:** Scores of pairwise alignments of the predicted amino acid sequences of Orf61 from six analyzed lambdoid phages: λ phage (NC_001416), Φ24_B_ phage (HM208303), 933W phage (NC_000924), VT2 Sakai phage (AP000422), Stx1 converting phage (NC_004913), and Stx2 converting phage II (NC_004914).

	λ	Φ24_B_	933W	VT2 Sakai	Stx1	Stx2_II
**λ**		70	70	70	70	70
**Φ24_B_**			100	100	100	100
**933W**				100	100	100
**VT2 Sakai**					100	100
**Stx1**						100
**Stx2_II**						

Pairwise scores are simply the number of identities between the two sequences, divided by the length of the alignment, and represented as a percentage. The multiple sequence alignment was performed using the ClustalW algorithm.
